# *TED-Face*: Texture-Enhanced Deep Face Reconstruction in the Wild

**DOI:** 10.3390/s23146525

**Published:** 2023-07-19

**Authors:** Ying Huang, Lin Fang, Shanfeng Hu

**Affiliations:** 1 Institute of Virtual Reality and Intelligent Systems, Hangzhou Normal University, Hangzhou 311121, China; fangmumu1995@gmail.com; 2 Department of Computer and Information Sciences, Northumbria University, Newcastle-upon-Tyne NE1 8ST, UK

**Keywords:** face reconstruction, texture enhancement, 3D morphable model, high fidelity, deep learning

## Abstract

We present *TED-Face*, a new method for recovering high-fidelity 3D facial geometry and appearance with enhanced textures from single-view images. While vision-based face reconstruction has received intensive research in the past decades due to its broad applications, it remains a challenging problem because human eyes are particularly sensitive to numerically minute yet perceptually significant details. Previous methods that seek to minimize reconstruction errors within a low-dimensional face space can suffer from this issue and generate close yet low-fidelity approximations. The loss of high-frequency texture details is a key factor in their process, which we propose to address by learning to recover both dense radiance residuals and sparse facial texture features from a single image, in addition to the variables solved by previous work—shape, appearance, illumination, and camera. We integrate the estimation of all these factors in a single unified deep neural network and train it on several popular face reconstruction datasets. We also introduce two new metrics, visual fidelity (VIF) and structural similarity (SSIM), to compensate for the fact that reconstruction error is not a consistent perceptual metric of quality. On the popular FaceWarehouse facial reconstruction benchmark, our proposed system achieves a VIF score of 0.4802 and an SSIM score of 0.9622, improving over the state-of-the-art Deep3D method by 6.69% and 0.86%, respectively. On the widely used LS3D-300W dataset, we obtain a VIF score of 0.3922 and an SSIM score of 0.9079 for indoor images, and the scores for outdoor images are 0.4100 and 0.9160, respectively, which also represent an improvement over those of Deep3D. These results show that our method is able to recover visually more realistic facial appearance details compared with previous methods.

## 1. Introduction

Reconstructing 3D faces from single-view images has received years of research attention in computer vision and computer graphics due to its broad applications in facial image editing, virtual reality [1], and entertainment. However, it remains a challenging problem as estimating facial geometry and appearance from a single image is inherently an inverse problem that may not admit a unique solution. Nevertheless, human eyes are particularly good at comparing a pair of faces (original and reconstructed) and judging their visual similarity, thereby serving as an oracle quantifying the quality of facial recovery solutions. One subtle yet powerful cue that influences human judgment is facial skin texture [2], which determines the interaction of lights and geometry to produce visual perception of faces.

**Figure 1:**
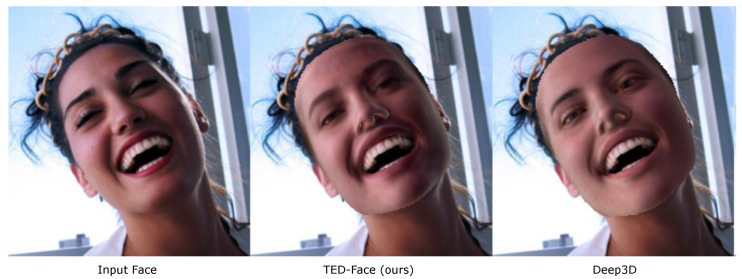
Reconstruction error does not equal visual similarity. While the root mean squared error (RMSE) between the input image (**left**) and our reconstructed output (**middle**) is 1.1677, which is slightly higher than that between the input and the Deep3D [17] output (**right**) of 1.1377, our result resembles the original face more closely in terms of skin tone and texture.

To address the ill-posed nature of image-based facial reconstruction, most of previous work represents both geometry and texture in low-dimensional spaces [3,4,5,6,7,8,9,10], in which the unique solution is found through minimizing the discrepancy between an input facial image and the rendering of an estimated face. This approach, albeit popular and effective in practice, can suffer from two main flaws. First, encoding spatially varying facial textures in a reduced space can incur information loss on high-frequency residuals around facial part boundaries and as a result lead to over-smoothed, plastic-like results. Those residuals, as shown in Section 3.4, are visually sparse and localized, which in theory cannot be captured using low-frequency basis functions of limited bandwidth (the uncertainty principle in Fourier analysis) [11]. Second, computer rendering of reconstructed faces as an essential step in deriving the reconstruction error to be minimized can bias the estimation because it acts as low-pass filters when a less sophisticated illumination model (e.g., with the spherical harmonic representation of lighting in existing work) is used for light transport computation [12]. This can produce faces lacking fine colors, too, as shown in Figure 1.

There are some algorithms that have been proposed to circumvent the limitation of low-dimensional facial texture models by using the UV space instead as a representation of facial textures [13,14,15]. However, estimating the dense UV map when a face is partially occluded is considerably unreliable, and learning the UV mapping of the whole head is not an easy task either [13,16,17]. Hence, the robustness issue of these methods can be a limiting factor when applied to unconstrained facial images in the wild.

We propose two simple yet effective techniques—***sparse texture enhancement*** and ***radiance residual learning***—to address the two aforementioned difficulties with existing methods separately. For the first issue, it arises because the standard pixel-to-pixel reconstruction error between input image and rendered face averages the contribution of each pixel’s discrepancy with equal weight and therefore fails to prioritize facial areas that exhibit sharp variations in color intensity. Indeed, it is thoroughly demonstrated in [18] that using image gradients to capture such first-order texture information is critical for high-quality image editing. Motivated by this, we propose to extract facial image gradients using the discrete Prewitt operator, which enables us to re-weight each pixel with its gradient magnitude for computing a texture-enhanced reconstruction loss to be jointly minimized with the standard one. Benefiting from the natural sparsity of image gradient intensities, the recovery algorithm can be effectively guided to produce sufficient details in high-frequency texture areas overlooked by previous work, thereby improving the visual appearance of generated faces.

The source of the second difficulty differs from the first one in that it concerns properly shading an estimated face to be rendered and compared with the input. Due to the low-pass nature of simplified illumination models, increasing the expressiveness of lighting functions may not be able to fully resolve this issue as there will always be residuals of radiance across the geometry of a facial shape. Drawing inspirations from [19], we propose to learn these residuals by directly regressing dense radiance compensations from the input image, which are then added to the base shading results for final rendering. While estimating full dense radiance without going through an analytic shading process can lead to serious over-fitting, our technique combines the strength of base shading and residual learning to recover more detailed facial colors.

**Figure 2:**
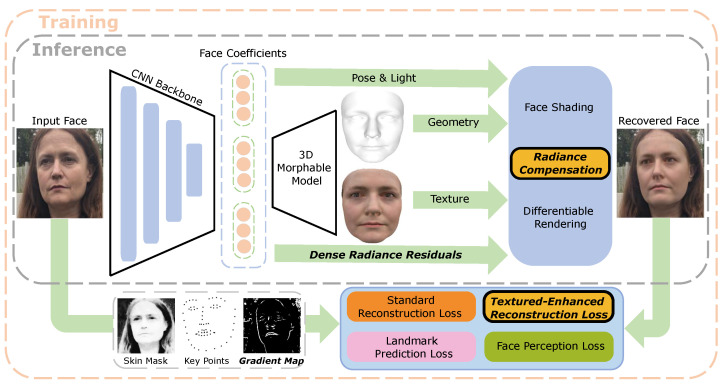
Overview of our TED-Face reconstruction system (Section 3.1). Given an input facial image, a deep convolutional neural network is used to predict the various coefficients of 3D face (Section 3.2), including the parameters of the coarse-grained 3DMM model (geometry, texture), illumination functions, face pose, and ***dense radiance residuals*** (Section 3.3). Multiple loss functions including our proposed ***texture-enhanced reconstruction loss*** (Section 3.4) are jointly used to train the whole system end-to-end.

We integrate the two above techniques into a unified deep learning architecture (Figure 2), along with the inference of facial geometry (using the 3D morphable model), facial texture, lighting functions, and camera parameters, for joint end-to-end training. We train and evaluate our system on a variety of public face reconstruction datasets, including CelebA [20], 300W-LP [21], I-JBA [22], FFHQ [23], LFW [24], and LS3D [25]. In addition to the commonly reported reconstruction error metric, we also adopt the structural similarity index (SSIM) [26] and visual fidelity index (VIF) [27] to quantify the similarity of original and recovered faces in a way that is more consistent with human judgment. The results show that our method consistently outperforms the state of the art on all evaluated datasets.

The contributions of this paper include the following:

- A new way to enhance the recovery of high-frequency facial textures by re-weighting facial reconstruction error using sparse image gradient intensities.
- A technique to directly learn dense radiance residuals for compensating the low-pass filtering of facial rendering.

The structure of this paper is as follows. We survey the existing methods for human face reconstruction in Section 2, through which we point out the unique advantage of our method for texture enhancement. We then describe our system in Section 3 and present the experimental results in Section 4. We conclude the paper in Section 5.

## 2. Related Work

### 2.1. 3D Morphable Model of Faces

To solve the inverse problem of 3D face reconstruction from single images, some low-dimensional parametric face models such as the 3D morphable model (3DMM) [3] have been proposed to constrain the solution process, playing a vital role in the field of vision-based face recovery and tracking. Early 3DMM typically construct linear low-dimensional facial geometry and texture spaces using a reduced number of eigen-basis functions from principal component analysis (PCA) decomposition on facial scan data [28,29,30,31,32]. Subsequent methods improve the model by using more training data and adding expression basis functions to the model for encoding faces of more variations [33]. While such a reduced model can greatly improve the efficiency and stability of face estimation and potentially reduce over-fitting, it is not easy to directly synthesize highly detailed, fast-changing facial texture variations, which can be a limiting factor in producing faces that agree with human judgment of visual similarity.

### 2.2. Deep Learning for 3DMM

Deep neural networks, as a powerful learning tool, have achieved tremendous success for vision-based 3D face reconstruction. In [34], they propose a model with encoder and decoder based on deep neural networks to directly learn the mapping from 2D images to 3D faces. Some works also use neural networks to learn the parameters of a 3DMM model for face recovery, which combines the advantage of neural networks in parameter fitting and the 3DMM model in recovering stable geometric and textural representations [6,7]. The advancement of deep learning solutions, however, comes with the requirement of more training data [35,36,37,38,39]. Due to the high cost of acquiring 3D face scanning data, some methods that rely on weekly-supervised learning have also been proposed to reconstruct faces through synthesis by analysis [6,9,17,34]. They generally work by projecting an estimated face back to an image plane via a differentiable rendering process and then comparing the rendered face with the original one to provide a supervision signal for training. Such an approach can effectively improve data efficiency and enhance the robustness of deep neural networks [40,41,42]. During training, these methods seek to minimize the reconstruction error that typically treats each facial location with equal importance, which can lead to over-smoothed estimation when the underlying low-dimensional face model (e.g., the 3DMM) has a limited representational capacity. We address this challenge by using image gradient intensities to re-weight each reconstruction location proportionally so that the learning process can be induced to focus more on numerically small yet visually prominent facial areas.

### 2.3. Facial Texture Representation

As the low-dimensional linear basis functions of 3DMM models can limit the visual fidelity of generated faces, techniques such as the UV mapping has been adopted to improve the quality of 3D face reconstruction [33,34]. In [33], they define an additional UV map on top of the 3DMM model to describe facial textures. The work of [34] implements an inverse projection from 2D image to 3D texture by constructing an UV space mapping relationship. In [43], a face texture extraction method via matrix factorization is proposed. While the method of [44] can directly regress depth values for each face pixel, it cannot recover the radiance intensity of color surface and requires an offline alignment step. In recent years, the neural radiance field (NeRF) has received a lot of attention [45,46,47,48]. Although the reconstruction fidelity is amazing, NeRF relies on voxel representation, which may not generalize well for face reconstruction. Computer rendering with a simplified shading model can limit the visual perception of estimated faces also through implicit low-pass filtering [12]. Inspired by the work of [19], we propose to directly regress radiance residuals from input image to be added on top of computer-generated face rendering. Albeit simple, we show that this technique works well for enhancing facial colors in reconstruction.

## 3. *TED-Face*: Our Proposed Approach

### 3.1. Overview of System

Our proposed system (Figure 2) is an end-to-end deep neural network architecture that accepts an input facial image (of arbitrary identity, pose, and expression) and estimates its corresponding 3D face model that contains geometry and texture as two essential outputs. During the testing phase, the input image is first processed by a backbone convolutional neural network (CNN) to extract deep features for estimating the 3DMM face parameters (geometry, texture), head pose orientation, lighting functions, and radiance residuals. Motivated by similar lines of work, we use the popular standard ResNet-50 [19] as our backbone network, which has sufficient capacity to extract texture-rich image features that are highly informative for facial recovery. These features are then used to infer the 3DMM parameters that allow us to reconstruct a full 3D textured face using the pre-learned geometry and texture basis functions. The face is subsequently shaded using the lighting functions and enhanced by the radiance residuals to be eventually rendered on the image plane with the estimated head pose. As the shading and rendering processes are both differentiable, the whole architecture can be trained provided with suitable loss functions. For training, we design the following losses:

- **Standard Reconstruction Loss**: measuring the pixel-to-pixel discrepancy between input and rendered faces with equal importance weight for each pixel.
- **Texture-Enhanced Reconstruction Loss**: calculating the same error as above but with an adaptive importance weight applied to each individual pixel using the corresponding image gradient intensity.
- **Landmark Prediction Loss**: measuring the distance from a set of detected 2D facial landmark points on the image and the corresponding set of estimated 3D key points after projection (using the head pose information).
- **Face Perception Loss**: computing the cosine dissimilarity between deep CNN feature embeddings (pre-trained for face recognition) of input and reconstructed facial images.

The two contributions of our architecture are the learning of radiance residuals for compensating for the shading process (Section 3.3), as well as re-weighting the standard reconstruction error with image gradient intensities to recover texture details (Section 3.4). The face model we use is detailed in Section 3.2, and the neural network architecture is presented in Section 4.1.

### 3.2. 3D Morphable Face Model

Following existing work, we adopt the 3D morphable model of faces [3] to represent the geometry and texture of a 3D face using linear combinations of their corresponding basis functions (i.e., subspace) as follows:

(1) $$\begin{array}{cc} S & =\bar{S}+B_{id}\alpha +B_{exp}\beta \end{array}$$

(2) $$\begin{array}{cc} T & =\bar{T}+B_{tex}\gamma \end{array}$$

where $\bar{S}$ and $\bar{T}$ are the mean face geometry and texture, respectively. The variations in geometry are encoded using a set of linear basis functions, $B_{id}$ and $B_{exp}$, which are constructed from PCA decomposition on a 3D face scan dataset to explain the identity and expression factors separately. Similarly, the variations in texture are compactly represented using the principal components $B_{tex}$ extracted from vertex colors of facial meshes on the same dataset. The unknown shape coefficients (α, β), which are to be inferred from an input facial image using our system (Figure 2), allow us to reconstruct a 3D face model using Equation (1). The texture parameters γ are estimated jointly with the geometry ones for facial color reconstruction using Equation (2). To instantiate the model, we use the popular Basel face space [33], which includes the pre-trained $\bar{S}$, $\bar{T}$, $B_{id}$, and $B_{t}$, with the latter two having 80 components (i.e., 80 dimensions). We estimate the facial expression subspace $B_{exp}$ from the FaceWarehouse dataset [49]. As a result, each 3D face without the ears and the neck part has 36,000 vertices.

### 3.3. Radiance Residual Learning

Once a 3D textured face is constructed using the technique in Section 3.2, the next step of our system is shading the face and then projecting it down to the image plane for comparison with the input. One of the contributions we make here is learning radiance residuals directly from the input image to compensate for the lack of fine details of shading.

**Face Illumination**. Following previous work, we assume that each face geometry is a Brownian surface (i.e., the reflected light is the same in all directions and does not absorb any incident light) and estimate face illumination using a set of spherical harmonic functions [50,51]. The radiance intensity at each vertex $s_{i}$ is calculated directly using its surface normal vector $n_{i}$ and the corresponding skin texture $t_{i}$ according to the following:

(3) $$\begin{array}{cc} \; & C\left(i|\sigma \right)=t_{i}\cdot \sum_{b=1}^{B^{2}}\sigma_{b}\Phi_{b}\left(n_{i}\right) \end{array}$$

(4) $$\begin{array}{cc} \; & \Phi_{b}:\;\mathrm{spherical}\;\mathrm{harmonic}\;\mathrm{basis}\;\mathrm{function} \end{array}$$

(5) $$\begin{array}{cc} \; & \sigma_{b}:\;\mathrm{spherical}\;\mathrm{harmonic}\;\mathrm{basis}\;\mathrm{coefficient} \end{array}$$

We choose *B* for three bands of frequency [9,17] to approximate lighting, resulting in nine spherical harmonic coefficients σ to be estimated from the input image for face shading.

***Radiance Compensation***. As indicated in Equation (3), the shading process is essentially a low-pass filter that only relies on several bands of low-frequency spherical harmonic functions while discarding the high-frequency components. This may not suffice for high-fidelity face reconstruction since fine facial texture colors cannot be adequately captured.

**Figure 3:**
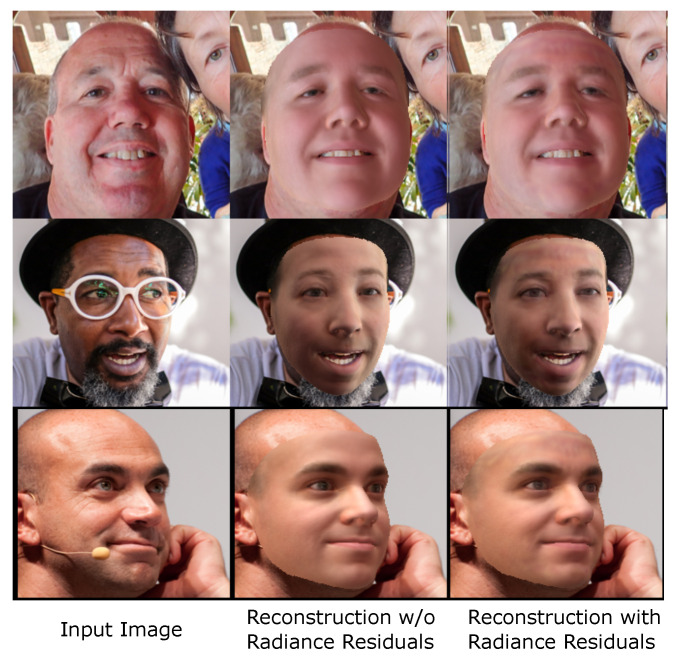
Radiance residuals enhance the visual fidelity of reconstructed faces. Estimating facial color appearance using a reduced-dimensional texture space and simplified shading can result in over-smoothed faces (**middle**) due to lack of fine details. Directly learning dense radiance residuals from an input image to compensate for the loss of details improves the appearance of the reconstructed faces (**right**).

We tackle this challenge using a deep residual learning [19] technique as follows:

(6) $$\begin{array}{c} \underset{\mathrm{radiance}\;\mathrm{compensation}}{\underset{︸}{L_{i}=C\left(i|\sigma \right)+\varphi_{i}}} \end{array}$$

in which $\varphi_{i}$ is the radiance residual we directly regress from the input image for each facial vertex *i*. We sum the base shading result C(i|σ) and the residual component $\varphi_{i}$ that cannot be reconstructed through shading alone to form the full estimation of face illumination. As shown in Figure 3, this technique can enhance the visual appearance of reconstructed faces in certain facial areas.

**Rendering**. Once shading is computed in (3) and compensated in (6), we use a camera model of perspective projection with an empirically chosen focal length for rendering an inferred 3D face on the image plane for comparison with the input facial image. The head pose parameters including a rotation matrix R∈SO(3) and a translation vector $t\in R^{3}$ are estimated from the input image [17], which needs to be applied on the 3D face before rendering. The projection process is differentiable and therefore permits continuous optimization of our system using gradient descent.

### 3.4. Sparse Texture Enhancement

As previewed in Section 3.1, our face reconstruction system requires several loss functions for training, among which the texture-enhanced reconstruction error is another contribution of this paper to enrich texture details.

**Standard Reconstruction Loss**. As our approach works in a weakly supervised synthesis-by-analysis manner, we need the standard reconstruction loss to measure how close an input facial image to the estimated face after shading and rendering for providing supervision signals [3,17]. To eliminate the effect of facial whiskers and hairs on face reconstruction, we use a pre-trained Bayesian classifier [17] to predict a face mask *M*, in which each element $M_{i}=\left\{\begin{array}{cc} 1 & ,if\;P_{i}>0.5\\ P_{i} & ,otherwise \end{array}\right.$ indicates the likelihood of the corresponding image pixel *i* belonging to facial skin, with $P_{i}$ being the raw probability output of the classifier. Now, we can calculate the pixel-to-pixel distance between an input face *I* and its rendered version $I^{\prime }$ as follows [3,6,9,52]:

(7) $$\begin{array}{c} L_{\mathrm{standard\_reconstruction}}=\frac{\sum_{i\in M}M_{i}\cdot {∥I_{i}-I_{i}^{\prime }∥}^{2}}{\sum_{i\in M}M_{i}} \end{array}$$

While the standard loss (7) widely adopted in existing work works well for bringing the estimated facial colors closer to the original ones *on average*, it does not differentiate between visually salient and non-salient areas, which can lead to quality loss of important facial texture details.

**Figure 4:**
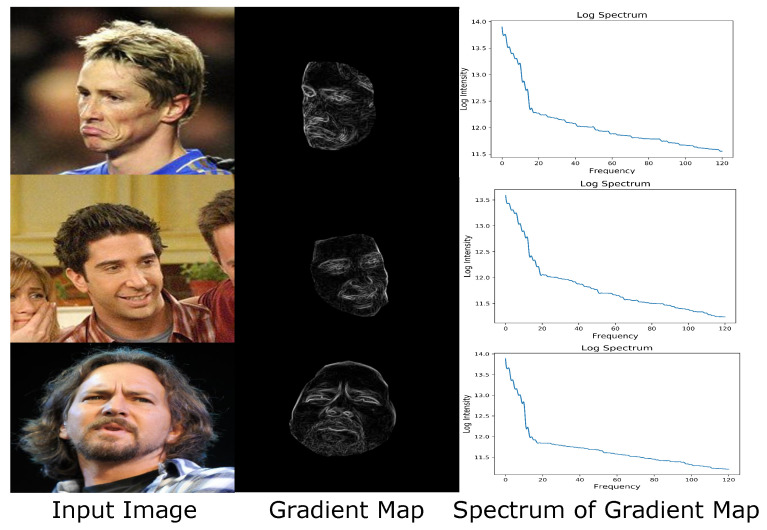
Image gradient magnitudes indicate high-frequency salient textures. Applying the 3×3 Prewitt convolutional kernel on the input image (**left**) produces the gradient map (**middle**, visualized as intensities) that more clearly highlight visually prominent areas such as the eyes, wrinkles, and nasolabial folds, producing consistent log spectrum shapes (**right**, zooming in to see details) that contain a large proportion of high-frequency components. We use these gradient intensities as importance weights in our system to guide the recovery of facial texture details.

***Texture-Enhanced Reconstruction Loss***. We propose to address the above-mentioned difficulty with the standard reconstruction loss by introducing a texture-sensitive importance weight for each pixel to more effectively guide the recovery process towards detail synthesis. Motivated by the success of gradient information for high-quality image editing [18], we adopt the simple yet effective Prewitt operator to compute image gradients and use their intensities to re-weight the reconstruction loss as follows:

(8) $$\begin{array}{c} L_{\mathrm{enhanced\_reconstruction}}=\underset{\mathrm{texture}\;\mathrm{enhancement}\;\mathrm{using}\;\mathrm{gradient}}{\underset{︸}{\frac{\sum_{i\in M}M_{i}\cdot {∥I_{i}-I_{i}^{\prime }∥}^{2}\cdot ∥\nabla_{i}∥}{\sum_{i\in M}M_{i}}}} \end{array}$$

where $\nabla_{i}$ is the 2D gradient vector (in both horizontal and vertical directions) at pixel *i* as derived from convolving the 3×3 Prewitt kernel with the input image *I*. It can be seen in Figure 4 that these importance weights clearly highlight visually prominent facial regions that undergo sharp texture variations, such as the eyes, wrinkles, nasolabial folds, and mouth. These areas contain relatively high-frequency contents, and we use those weights to prioritize their recovery, which can produce faces of higher fidelity.

**Landmark Prediction Loss**. To improve the geometric alignment of input and reconstructed faces, we also introduce the landmark prediction loss function that penalizes the deviation of projected 3D key facial points from their ground-truth counterparts on the input image. We use a state-of-the-art facial landmark detection method to localize 68 key points on a face image [6,9,17]. We then track the indices of those points on the 3D face model to obtain their positions on the 2D image after projection for loss calculation:

(9) $$\begin{array}{c} L_{\mathrm{landmark\_prediction}}=\frac{1}{N}\sum_{n=1}^{N}\omega_{n}{∥v_{n}-v_{n}^{\prime }∥}^{2} \end{array}$$

where N=68 is the number of landmark points, $v_{i}$ corresponds to the *i*-th point on the input image, and $v_{i}^{\prime }$ is the 2D projection of the corresponding 3D key point on the face model. We set the weight $\omega_{i}$ to 20 for points at visually sensitive facial areas, such as the mouth and nose, while weighting the remaining points with 1.

**Face Perception Loss**. In addition to the above losses that work in the raw image domain, we also incorporate a perceptual loss that can enforce the specificity of generated faces by pulling their feature representations closer to that of the input in a semantically meaningful space. To this end, we employ a pre-trained face recognition network FaceNet [53] to extract deep identity-relevant features for input and rendered faces, giving rise to the following loss:

(10) $$\begin{array}{c} L_{\mathrm{face\_perception}}=1-\frac{\langle f\left(I\right),f\left(I^{\prime }\right)\rangle }{∥f(I)∥\cdot ∥f(I^{\prime })∥} \end{array}$$

where f(∗) denotes the feature extraction function of FaceNet and $\langle *,*\rangle$ computes the inner product of two feature vectors. Since the features were originally learned for solving identity recognition, the perception loss (10) can enhance the visual similarity of original and recovered faces.

**Total Loss**. Summing up all loss functions together, we arrive at the following total loss that includes an additional parameter regularization term to prevent degradation of facial geometry and texture recovery:

(11) $$\begin{array}{cc} L_{\mathrm{total}}= & \omega_{s}L_{\mathrm{standard\_reconstruction}}+\\ \; & \omega_{e}L_{\mathrm{enhanced\_reconstruction}}+\\ \; & \omega_{l}L_{\mathrm{landmark\_reconstruction}}+\\ \; & \omega_{f}L_{\mathrm{face\_perception}}+\\ \; & \underset{\mathrm{parameter}\;\mathrm{regularization}\;\mathrm{term}}{\underset{︸}{\omega_{\alpha }{∥\alpha ∥}^{2}+\omega_{\beta }{∥\beta ∥}^{2}+\omega_{\gamma }{∥\gamma ∥}^{2}}} \end{array}$$

where $\left\{\omega_{s},\omega_{e},\omega_{l},\omega_{f},\omega_{\alpha },\omega_{\beta },\omega_{\gamma }\right\}$ are the weights for each loss term that need to be chosen.

## 4. Experimental Results

### 4.1. Implementation Details

The backbone convolutional neural network of our system is the standard ResNet-50 network [19], which we modify to allow its final fully connected layer to produce the following predictions from an input facial image *I* (cropped and resized to 224×224 in our experiments):

- α: 80 identity coefficients of facial geometry in (1);
- β: 80 expression coefficients of facial geometry in (1);
- γ: 80 coefficients of facial texture in (3);
- σ: 9 spherical harmonic coefficients in (3);
- *R*: 9 parameters in a 3×3 rotation matrix in Section 3.3;
- *t*: 3 translation amounts in Section 3.3;
- φ: 3×36 k radiance with one for each vertex in (6).

We train our system using the Adam optimizer [54] with the default learning rates. Our network is initialized by the model pre-trained on the ImageNet dataset [55]. The weight for each loss term in (11) is set as $\omega_{s}=1.92$, $\omega_{e}=1.92$, $\omega_{l}=1.6e-3$, $\omega_{f}=0.2$, $\omega_{\alpha }=1.0$, $\omega_{\beta }=0.8$, and $\omega_{\gamma }=10$. The epochs and batch size are set to 20 and 16, respectively. We find that these hyper-parameters work well in practice. Our training and testing are performed on a PC with an i9 9900k CPU, 32GB RAM, and an 2080ti graphics card. It is noted that in [56] the authors propose the use of intermediate deep features for object representation, which can be cheaper to compute and therefore enable faster inferences. This inspires us to explore in future work a similar idea to speed up facial reconstruction, especially for real-time applications.

### 4.2. Datasets and Evaluation Metrics

We evaluate our proposed face reconstruction method on six widely used facial image datasets, namely CelebA [20], 300W-LP [21], I-JBA [22], FFHQ [23], LFW [24], and LS3D [25]. These datasets contain a large quantity of facial images of a wide-range of identifiers, expressions, and poses, which are sufficiently diverse and challenging to benchmark different methods. Following previous work, we balance the pose and race distributions to randomly select 320K face images for training and 6K images for testing.

**Evaluation Metrics**. For the evaluation of facial geometry reconstruction, we use the root mean squared error (RMSE) between two rigidly aligned faces (one is input and the other is estimation) to measure the geometric reconstruction error. For the comparison of visual fidelity, we utilize the structural similarity index (SSIM) [26] and visual fidelity index (VIF) [27], which are both popular metrics for image quality assessment that take human perception into account, in contrast to pixel-level reconstruction errors that are weakly correlated with human judgment of visual resemblance.

In the following, we first compare our method with several state-of-the-art methods on recovering facial color appearance (Section 4.3) and then present evaluation results on the quality of estimated 3D facial geometry (Section 4.4). Afterwards, we show ablation study results to validate the effectiveness of our two key design choices (Section 4.5): sparse texture enhancement and radiance residual learning.

### 4.3. Evaluation of Facial Appearance Reconstruction

**Controlled Scenarios**. Here, we compare our method with several state-of-the-art methods for face reconstruction including Deep3D [17], Detailed 3D [57], MGCNet [58], and Face Rec [59], on the FaceWarehouse dataset [49] that was collected under well-controlled settings. In Table 1, we use the 300 images from the FaceWarehouse dataset [49] as the validation set and calculate the SSIM and VIF scores between the original and reconstructed facial images. The results show that our method improves upon the competitors on both metrics. Particularly, the VIF index is improved by over 6% compared with the score obtained by Deep3D, which is the second highest in the table.

**Figure 5:**
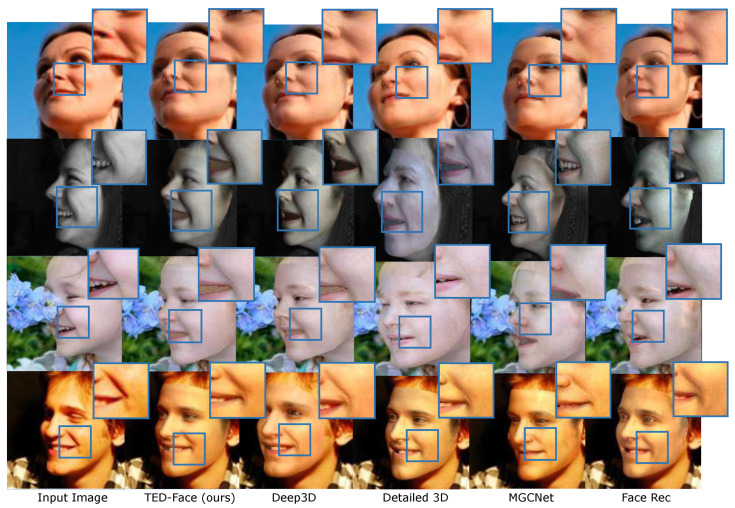
Comparison with state-of-the-arts on the low-resolution AFLW-2000 test set [21]. From left to right are the input faces, our reconstruction results, and the faces recovered by Deep3D [17], Detailed 3D [57], MGCNet [58], and Face Rec [59]. Certain facial areas are zoomed in to highlight the improvement of our method for reconstructing fine facial texture details.

**Low-Resolution Images**. Figure 5 shows the comparison with state-of-the-art methods on the low-resolution AFLW-2000 facial image dataset [21]. Due to the lack of high-frequency image information, these methods struggle with estimating accurate facial texture and illumination details, leading to unsatisfactory reconstruction results in some highlighted areas. In contrast, our method alleviates the challenge of low-resolution inputs by guiding texture reconstruction using image gradients and enhancing lighting with estimated radiance residuals. This gives rise to visually clearer and higher-quality recovered faces.

**Figure 6:**
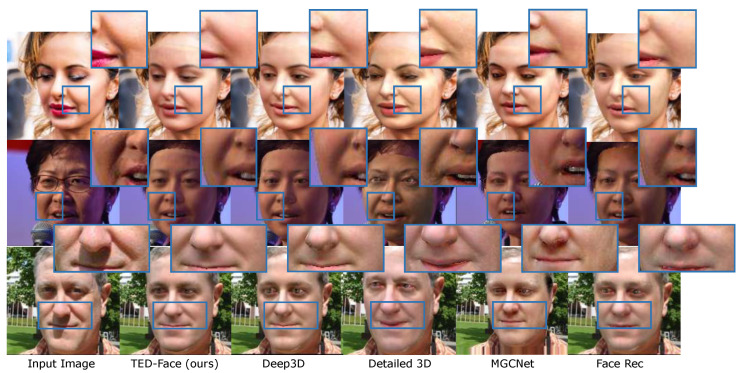
Comparison with state-of-the-art methods on the high-resolution FFHQ dataset [23]. From left to right are the input faces, our reconstruction results, and the faces recovered by Deep3D [17], Detailed 3D [57], MGCNet [58], and Face Rec [59]. Certain facial areas are zoomed in to highlight the improvement of our method for reconstructing fine facial texture details.

**High-Resolution Images**. Similarly, we perform evaluation and comparison on high-resolution facial images. Figure 6 shows our validation results on the high-resolution FFHQ (1024 × 1024) dataset. In contrast to the AFLW-2000 facial image dataset [21], the FFHQ dataset consists of facial images that contain more accurate appearance and scene illumination cues. Benefiting from the texture enhancing features of our method, it can be seen that our reconstruction around facial skin folds matches that of the input images much more closely in comparison with the results generated by the other methods. This, together with the low-resolution evaluation, shows that our method is able to process both poor and high-quality input images, which can be desirable for applications that involve both low and high-end face capturing devices.

**Figure 7:**
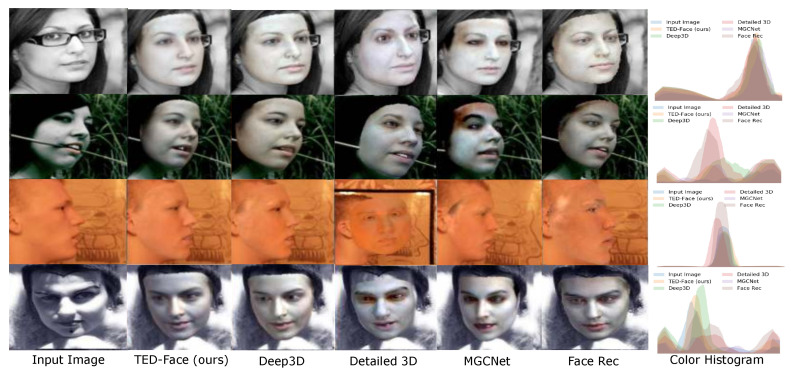
Comparison with state-of-the-art methods on the indoor-outdoor LS3D-300W validation dataset [25]. From left to right are the input faces, our reconstruction results, and the faces recovered by Deep3D [17], Detailed 3D [57], MGCNet [58], and Face Rec [59]. The last column shows the comparison of color histograms of facial images produced by these methods (zoom in to see details).

**Indoor-Outdoor Scenes**. We also conducted experiments on the LS3D-300W face dataset [25], which contains facial images captured in both indoor and outdoor environments. Noted that all the compared methods in our study have been replicated, and there is no case of copying the results of other papers. The results in Table 2 were obtained using the LS3D-300W dataset as the test set, which contains many low-resolution facial images captured indoors and outdoors. If the test set is input into the Detailed 3D method, due to the low quality of the images, there are many cases where it cannot generate a facial mesh or obtain corresponding facial textures, so Detailed 3D is not included in Table 2 as a comparison method. As shown in Table 2, our method makes considerable improvements upon the state-of-the-art methods on both the SSIM and VIF evaluation metrics for indoor and outdoor scenes. We show the visual comparison of reconstructed faces in Figure 7, along with the comparison of image color histograms focused on the facial areas. Despite the variety of the scene lighting conditions, it can be seen that our method produces more visually accurate facial texture representations compared with the other methods. By inspecting the color histograms, it is also validated that our estimated facial color distributions are numerically closer to that of the input images.

### 4.4. Evaluation of Facial Geometry Reconstruction

Our method focuses on enhancing the final visual presentation of reconstructed faces (as measured by the SSIM and VIF metrics) while ensuring that the quality of generated 3D face geometric models is on par with that of the current state-of-the-art methods in terms of geometric reconstruction error. To this end, we further conducted experiments on two face scan datasets: the MICC Florence 3D face dataset [60] and the FaceWarehouse dataset [61], both of which contain ground-truth facial scan data. The MICC dataset contains 53 subjects and 3 video sequences captured in well-controlled, indoor and outdoor scenes. For FaceWarehouse, we use nine subjects with 20 expressions for evaluation.

**Figure 8:**
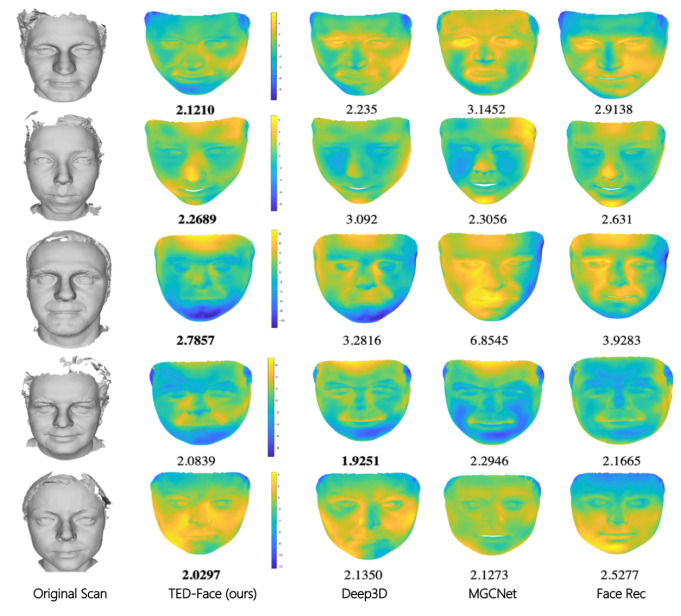
Visualization of facial geometry reconstruction errors. From left to right are the original facial scan, our reconstruction result, and the face recovered by Deep3D [17], MGCNet [58], and Face Rec [59]. The warmer the color visualization of a point, the higher the corresponding geometry reconstruction error. The score underneath each image is the root mean squared error between the reconstructed face and the original facial scan after rigid registration.

In Table 3, we present the reconstruction error results for the 3D face models generated using our and the state-of-the-art methods, among which our baseline is the Deep3D method [17]. In recent years, various methods based on neural radiance fields have been proposed for face reconstruction. However, these methods are sensitive to data and have limitations in the scope of application scenarios, making them unable to serve as a general solution for parameterized face modeling. Parametric models are still the main solution for general face modeling. This paper mainly aims to address the issue of insufficient expressive power of parametric face models. Therefore, the methods we used for comparison are also based on the 3DMM parametric face model. Currently, the state-of-the-art method for 3D face reconstruction based on 3DMM is still Deep3D. Most researchers in the field of 3DMM-based research compare their methods with Deep3D. Other parametric face model methods are different from the 3DMM approach in terms of the construction of the face parameter structure. We only compare methods within the same class to demonstrate the performance improvement of the proposed method. It can be seen that the accuracy of facial geometries estimated by our method is similar to that generated by Deep3D while outperforming MGCNet [58], Face Rec [59], and Detailed 3D [57] to a large extent. Figure 8 visualizes the geometric errors of face reconstruction results generated by each method using heat map, with the error metric displayed below each rendered image. We omit the results of Detailed 3D to avoid color map distortion because they are not comparable with the others due to their much larger errors. It can be seen that our method maintains a similar quality of facial geometry estimation as that of Deep3D. In the meantime, the enhancement of visual appearance of reconstructed faces brought by our method is visually clear and numerically large, as experimentally validated in Section 4.3.

### 4.5. Evaluation of Design Choices

**Figure 9:**
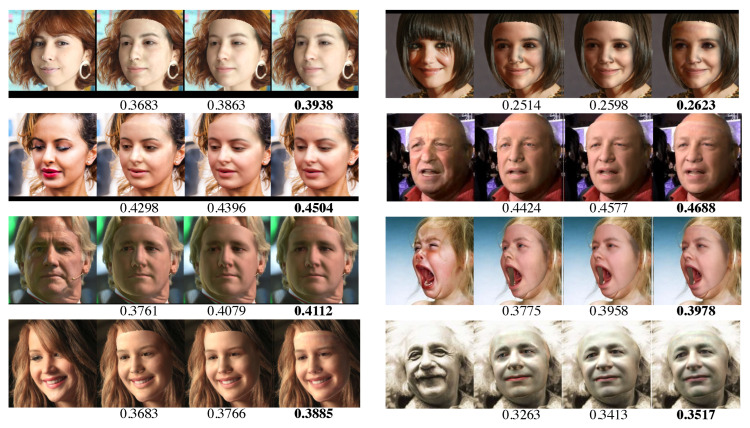
Comparison of reconstructed faces using different method configurations of ours. The images from left to right within each group correspond to the input face, baseline result, result with sparse texture enhancement (STE, key design choice 1, Section 3.4), and result with both sparse texture enhancement and radiance residual learning (RRL, key design choice 2, Section 3.3). The VIF score for each reconstructed face is shown underneath (ref. Table 4).

To validate the effectiveness of our design choices, we also perform ablation studies on the FaceWarehouse dataset [49] using 150 different individuals, each with 20 varying expressions. Table 4 shows the effects of adding each module design. The results indicate that our image-gradient-guided texture enhancement method significantly improves the final visual fidelity of reconstructed faces, while the radiance compensation technique further enhances the visual representation of the results. Figure 9 presents several visual and quantitative comparisons of faces generated using our system with different design choices. It can be see that our full system with both texture enhancement and radiance compensation produces the highest-quality face recovery results, compared with the baseline versions.

## 5. Conclusions and Future Work

We have proposed a new method, *TED-Face*, for reconstructing 3D facial geometry and texture from singe view images using deep learning. Our method works in an analysis-by-synthesis manner similar to previous work on vision-based 3D face reconstruction while improving over them on two important technical aspects to produce faces of higher visual fidelity. Accordingly, we have made two main contributions in this paper:

- We have proposed a new, simple yet effective technique, sparse texture enhancement, that can guide the inverse estimation process to learn to recover high-frequency facial texture details by re-weighting the standard per-pixel reconstruction loss with image gradient intensities extracted using the Prewitt operator. This was inspired by the Poisson image editing approach of [18], and it was shown in Table 4 to enhance the visual quality of estimated faces as measured by the SSIM and VIF metrics.
- We have also proposed a new technique named radiance residual learning, which can regress dense radiance residuals from input images to fill in facial appearance details that cannot be reproduced by a reduced texture space and shading model. The technique is straightforward to implement on top of facial reconstruction methods (including ours) that use the 3DMM model for surface estimation and shading. The results in Table 4 show that it further improves the visual quality of recovered faces.

We have evaluated our proposed system on two popular facial recovery datasets: FaceWarehouse [49] and LS3D-300W [25]. The results show that on the former, our system achieves a VIF score of 0.4802 and an SSIM score of 0.9622, improving over the state-of-the-art Deep3D method [17] by 6.69% and 0.86%, respectively. On the latter, our system obtains a VIF score of 0.3922 and an SSIM score of 0.9079 for indoor images, and the scores for outdoor images are 0.4100 and 0.9160 respectively, which also represent an improvement over that of Deep3D.

Due to the use of the low-dimensional 3DMM model for facial representation, our method has certain limitations that may prevent it from recovering accurate faces in challenging scenarios. First, the 3DMM model assumes a linear subspace of facial geometry, which greatly alleviates the difficulty of the inverse estimation problem but can fail to capture fine facial geometric details (e.g., wrinkles due to aging). As shown in the second column of Figure 8, the geometric reconstruction errors around the nose and mouth regions are generally higher than that in other flat areas. Second, our method uses spherical harmonic functions to approximate the facial shading process (Equation (3)). This process is differentiable and therefore enables gradient-based optimization. However, it can introduce approximation errors when the ground-truth lighting condition is overly complex and cannot be captured using a limited set of basis functions. Also, the shading is only calculated based on the surface normals and textures of a face as in the 3DMM model, which means that it cannot reproduce any sub-surface scattering phenomenon that can often be found when thin skins are lit by strong directional lights.

To address the aforementioned shortcoming of the 3DMM surface shading component, as an important line of future work, we plan to replace the currently used facial texture model with a physically more realistic one that can account for more intricate illumination effects such as subsurface scattering. The new model may contain a layered structure that represents the heterogeneous properties (e.g., albedo, scattering coefficient, phase coefficient) of skin, muscle, and fat, respectively, each of which would account for different illumination effects. This would allow us to reproduce facial appearance with richer details as human skin is not entirely opaque as implied by the 3DMM representation, especially when the skin is thin and the lighting is strongly directional. Differentiable volume rendering coupled with Monte Carlo sampling techniques for sub-surface lighting calculations can be a promising solution to this goal.

Due to the prevalent applications of facial reconstruction in virtual reality and mobile platforms, it is important that the reconstruction process can be run in real-time to account for dynamic contents. Our system is deep-learning-based and can be run quickly on powerful graphics cards. However, it is still heavy for commodity platforms, and, as a result, we intend to conduct further research to reduce the computational overhead via network pruning and quantization. While we have conducted experiments on several popular datasets in this work, the generalization ability of our method compared to other datasets remains to be seen, especially for human faces in the wild under challenging lighting conditions. Application to video face datasets is also worth consideration given their dynamic nature. Another line of future research is exploring data augmentation techniques to improve the generalization performance of trained models. One example could be using neural radiance field methods to relight facial images to create more diverse training data, which we plan to investigate.

## Figures and Tables

**Table 1:** Comparisons with state-of-the-art methods on the FaceWarehouse dataset [49]. Our method outperforms these methods on both VIF and SSIM metrics that measure visual fidelity of reconstructed facial images.

Method	VIF ↑	SSIM ↑
MGCNet [58]	0.4150	0.9359
Face Rec [59]	0.4462	0.9544
Detailed 3D [57]	0.4497	0.9561
Deep3D [17]	0.4501	0.9540
*TED-Face* (ours)	**0.4802**	**0.9622**

**Table 2:** Comparisons with state-of-the-art methods on the indoor-outdoor LS3D-300W dataset [25]. Our method outperforms these methods on both VIF and SSIM metrics that measure visual fidelity of reconstructed facial images.

Method	VIF↑	SSIM↑
	Indoor/Outdoor	Indoor/Outdoor
MGCNet [58]	0.3545/0.3581	0.8993/0.9060
Face Rec [59]	0.3661/0.3799	0.8998/0.9120
Deep3D [17]	0.3712/0.3871	0.8953/0.9052
*TED-Face* (ours)	**0.3922/0.4100**	**0.9079/0.9160**

**Table 3:** Comparison of facial geometry reconstruction errors. We use the iterative closest point (ICP) algorithm to align original and reconstructed 3D faces for computing the mean and standard deviation of point-to-point distances (mm) between the two on the MICC Florence 3D face dataset [60] and the FaceWarehouse dataset [61].

Method	Florence	FaceWarehouse
	Mean/Std ↓	Mean/Std ↓
MGCNet [58]	5.7375/8.1042	0.0313/0.332
Face Rec [59]	5.0877/6.6775	0.0295/0.2999
Detailed 3D [57]	2.4551/2.1502	0.0157/0.0126
Deep3D [17]	**1.7038**/2.0680	0.0149/0.0123
*TED-Face* (ours)	1.7065/**2.0647**	**0.0140/0.0120**

**Table 4:** Ablation study results of our method on the FaceWarehouse dataset [49]. The two key design choices we propose in this paper, namely sparse texture enhancement (STE) and radiance residual learning (RRL), improve the visual fidelity of reconstructed faces compared with the baseline that does not have those modules.

Configuration	VIF ↑	SSIM ↑
Baseline	0.4599	0.9622
Baseline+STE	0.4828	0.9670
Baseline+STE+RRL (our full model)	**0.4903**	**0.9692**

## Data Availability

The datasets used in this study can be obtained from links https://mmlab.ie.cuhk.edu.hk/projects/CelebA.html, https://www.tensorflow.org/datasets/catalog/the300w_lp, https://github.com/NVlabs/ffhq-dataset, http://vis-www.cs.umass.edu/lfw/ (accessed on 13 June 2023).
